# Toxic and repellent effects of hemp leaf extracts and cannabidiol against adult female yellow fever mosquitoes, *Aedes aegypti*

**DOI:** 10.1371/journal.pone.0354696

**Published:** 2026-09-15

**Authors:** Erick J. Martínez Rodríguez, P. Larry Phelan, Luis Canas, Nuris Acosta, Paola N. Feliciano, Jafina Tubbs, Eric Loperena, Harinantenaina L. Rakotondraibe, Peter M. Piermarini

**Affiliations:** 1 Department of Entomology, The Ohio State University, Wooster, Ohio, United States of America; 2 Universidad de Puerto Rico, Aguadilla, Puerto Rico, United States of America; 3 Department of Medicinal Chemistry and Pharmacognosy, The Ohio State University, Columbus, Ohio, United States of America; University of Messina, ITALY

## Abstract

Hemp (*Cannabis sativa*) derived products have emerged as potential sources of novel biopesticides. We recently demonstrated that a hemp leaf extract was toxic to larvae of the yellow fever mosquito *Aedes aegypti*, and that cannabidiol (CBD) was the primary larvicidal component of the extract. Here we evaluated the toxic and repellent effects of a hemp leaf extract and CBD against adult female *Ae. aegypti*. Toxicity was assessed using an acute topical assay in which mosquitoes received a 500 nL application to the thorax and mortality was recorded at 24 h. Repellency was then evaluated using two complementary bioassays: (i) a no-choice membrane blood-feeding assay to quantify feeding deterrence, and (ii) a mosquito airborne repellency test (MART) to assess spatial repellency. Topical application of CBD-rich hemp leaf extract induced dose-dependent mortality in adult *Ae. aegypti* with a LD_50_ of 22.13 µg/mosquito at 24 h. When CBD isolate was tested at its equivalent doses in the extract, it caused significantly lower mortality than the hemp extract. In the membrane blood-feeding repellency assay, the hemp leaf extract repelled mosquitoes in a dose-dependent manner, whereas CBD produced weaker effects even at higher equivalent doses. In the MART assay, the hemp extract did not elicit spatial repellency, suggesting low volatility of the repellent compounds. Taken together, our results demonstrate that CBD does not fully explain the adulticidal or repellent effects of the CBD-rich hemp leaf extract against adult female *Ae. aegypti* suggesting that other compounds contribute to the bioactivity of hemp leaf extracts against adult female mosquitoes.

## Introduction

The yellow fever mosquito *Aedes aegypti*, is the principal vector of medically important arboviruses, such as chikungunya, dengue and yellow fever [1–5]. These diseases altogether affect hundreds of millions of people annually, causing significant morbidity and mortality worldwide, particularly in tropical and subtropical regions [6]. Vector control is the primary strategy for reducing transmission and disease burden for most of these arboviruses due to the absence or limited efficacy of vaccines and/or therapeutics [7]**.** Insecticides, like pyrethroids, have been essential for mosquito control, but their overuse has led to metabolic and/or target-site resistance in *Ae. aegypti* populations worldwide, compromising current chemical control strategies [8–11]. Thus, discovery of insecticidal compounds with novel modes of action is needed for improving mosquito control.

Biopesticides derived from plant secondary metabolites are potential alternatives to pyrethroids [12–14]. These natural products are composed of chemically diverse defense compounds that plants deploy against herbivores. Many secondary metabolites exhibit insecticidal, antifeedant, and/or repellent properties against arthropods that can potentially be leveraged for pest control [15]. Moreover, complex blends of compounds found in raw extracts or essential oils of plants can target multiple biochemical mechanisms, potentially reducing the chances for resistance to evolve [16].

A plant that has recently been recognized as a potential source of novel insecticides is hemp, *Cannabis sativa* L*.*, which produces a diverse array of secondary metabolites, including over 100 terpenes and more than 90 phytocannabinoids [17]. These compounds are synthesized and stored in glandular trichomes, are most concentrated in leaves and flowers, and have been hypothesized to function in chemical defense against arthropod herbivores [15,17]. A number of the terpenes produced by hemp (such as myrcene, limonene, pinene, linalool, caryophyllene, among others) are also found in plants with demonstrated contact toxicity and repellency against a wide range of insect pests [18,19]. Additionally, over 20 flavonoids are found in *C. sativa* and are known to be toxic or repellent to various pests [17,20–22].

In mosquitoes, studies examining the toxic effects of hemp-derived products against larvae (e.g., *Culex quinquefasciatus*, *Culex pipiens*, *Aedes albopictus*, and *Ae. aegypti*) and adults (*Cx. quinquefasciatus*) have attributed the toxicity to terpenes, based on their high abundance in hemp [18,23–27]. Phytocannabinoids have received less attention for their potential toxicity against mosquitoes, but a recent study by our group demonstrated cannabidiol (CBD) was the primary toxic compound responsible for larvicidal activity of a hemp leaf methanolic extract against 1^st^ instar *Ae. aegypti* [28]. Moreover, we found that a CBD isolate showed similar larvicidal potency against pyrethroid-susceptible and pyrethroid-resistant *Ae. aegypti*, suggesting that CBD operates via a novel mode of action compared to pyrethroids [28]. In the present study, we build on our previous study by evaluating the toxic and repellent effects of the same hemp leaf methanolic extract and a CBD isolate against adult female *Ae. aegypti*.

## Materials & methods

### Hemp plants and extract preparation

We used the same hemp leaf extract from our previous study [28]. In brief, hemp plants (variety Tango Kush) were maintained in 38 L plastic pots filled with Pro-Mix BX soilless medium (Premier Tech, Rivière-du-Loup, QC, Canada) in an environmentally controlled greenhouse at 25°C and 16h:8h (light:dark) photoperiod (Argus Control System, Conviron, Langley Twp, BC, Canada). Hemp leaves were harvested seven months after planting, air-dried at 25 °C for seven days, and ground into powder. The powdered material (150 g) was extracted in 4 L of methanol (99.9%) for three weeks at 20 °C with daily agitation, followed by filtration and methanol removal using a rotary evaporator (Heidolph NA, Wood Dale, IL, USA) at 30 °C and 65 rpm. The resulting crude extract was stored at 4 °C and resuspended in 100% acetone on the day of bioassays. We previously demonstrated that CBD accounts for approximately 20% (w/w) of the dried mass of this hemp leaf extract, as determined by gas chromatography–mass spectrometry (GC–MS) [28].

### *Ae. aegypti* colonies

Adult females of *Ae. aegypti* (Liverpool strain LVP-IB12, MRA-735, contributed by David W. Severson) were reared from eggs using established methods [28,29]. In brief, larvae were fed daily with 1 tablet of fish food (Tropical Tablets, Tetramin, Blacksburg, VA, USA). Adult mosquitoes were fed 10% sucrose *ad libitum*. For egg production, adult females were fed defibrinated rabbit blood (Hemostat Laboratories, Dixon, CA, USA) using a membrane feeder (Hemotek, Blackburn, UK). All mosquitoes were reared in environmentally controlled chambers (Percival Scientific, Perry, IA, USA) at 28 °C and 80% relative humidity, with a 12h:12h (light:dark) photoperiod.

### Topical toxicity bioassays on adult female *Ae. aegypti*

Topical toxicity on adult female mosquitoes was measured using a previously described bioassay [30]. In brief, groups of 10 adult female mosquitoes (4–7 days post-emergence, non-blood fed) were immobilized on ice and then 500 nl of 99.7% acetone (solvent control), a hemp leaf extract or CBD isolate (both dissolved in 99.7% acetone) was applied to the dorsal thorax with a repeating dispenser (PB600−1, Hamilton, Reno, NV). For the hemp leaf extract, concentrations of 100, 50, 25, 12.5, 6.25, 3.12 mg/mL were used, which delivered doses of 50, 25, 12.5, 6.25, 3.12, and 1.56 µg/mosquito. For the CBD isolate (99% purity, Samson Extract, Geneva AL, USA), two concentrations (20 and 10 mg/mL) were used, which delivered doses of 10 and 5 µg/mosquito, respectively. Immediately after treatment, the mosquitoes were transferred to 32 oz. containers (Ziploc, Bay City, MI) with screened lids and cotton soaked in 10% sucrose. The containers were held under normal rearing conditions and mortality was assessed at 24 h after treatment. Mortality was corrected for solvent effects using Abbott’s formula [31]. If solvent mortality exceeded 20% then the experiment was discarded.

### Repellency bioassays on adult female *Ae. aegypti*

#### No-choice membrane blood-feeding bioassay.

Feeding disruption was evaluated using a no-choice membrane blood-feeding assay [30]. In brief, 24 h prior to an experiment, groups of 20 adult female mosquitoes (4–7 days post-emergence) were placed in small cages (17.5 x 17.5 x 17.5 cm, Bug Dorm, MegaView Science Co., Ltd, Taiwan) with cotton soaked in water. At the start of the experiment, the cotton was removed and a feeding disc (5.7 cm²) of a Hemotek membrane feeder (Blackburn, UK) was added. The disc was warmed to 37°C and prepared as described below. A collagen membrane (Hemotek) was placed on the surface of a feeding disc and the membrane was then covered with a thin nylon fabric (No nonsense Regular Pantyhose, Kayser-Roth Corporation, Greensboro, NC). The feeding disc was then filled with defibrinated rabbit blood (HemoStat Laboratories, Dixon, CA) containing approximately 2 mg of adenosine 5’-triphosphate (ATP) per mL of blood, as a feeding stimulant. The nylon fabric was then treated with either 250 μL of hemp leaf extract dissolved in acetone, CBD isolate dissolved in acetone, or 100% acetone (solvent control). For the hemp leaf extracts, concentrations of 3.6, 11, 33, and 100, mg/mL were used, which resulted in an application of 0.15, 0.47, 1.42, and 4.33 mg/ cm^2^. For the CBD isolate, concentrations of 2.5, 5, 10, 12.5, 25, 50 and 100 mg/mL were used, which resulted in an application of 0.11 0.21, 0.433, 0.55, 1.08, 2.16, 1.08, 2.16, and 4.33 mg/cm^2^. The feeding disc was introduced to a cage of mosquitoes in an environmentally controlled rearing chamber held at normal reading conditions. After 1 h, the disc was removed and mosquitoes were cold immobilized at 4 °C for 15 min. Mosquitoes were then visually inspected for blood to determine whether they fed. Any mosquitoes considered unfed were confirmed to not contain trace amounts of blood by crushing their abdomen on white filter paper. Unfed mosquitoes were considered repelled from the blood source. Percent repellency was calculated by using the following formula:


$$\begin{array}{c} \%\text{ Repellency}=\frac{\left({\%\text{ Fed}}_{\text{Control}}-{\%\text{ Fed}}_{\text{Treatment}}\right)}{{\%\text{ Fed}}_{\text{Control}}}\times \text{1}\text{0}\text{0} \end{array}$$

If fewer than 70% of the solvent control mosquitoes fed on blood, then the experiment was discarded.

#### Mosquito airborne repellency test (MART).

Non-contact repellency was determined using MART, following a protocol similar to Kwon et al [32]. Twelve non-blood fed adult female mosquitoes (4–7 days old) were transferred to a 18 cm plastic tube formed by connecting two vials (28.5 × 95 mm; VWR International, Radnor, PA) with the closed ends removed to create an open passage between chambers. The open ends of the tubes were covered with circular pieces of wire mesh (2.5 cm) to prevent mosquitoes from escaping and making contact with chemical treatments. For each assay, 50 µL of either acetone or hemp leaf extract (in acetone) was applied to a 2.5-cm diameter filter paper (Whatman, Buckinghamshire, UK). For the hemp leaf extract, concentrations of 3.6, 11, 33, and 100 mg/mL were used, which in an application of 0.03, 0.11, 0.33, and 1.01 mg/cm^2^. Treated filter papers were placed in a fume hood for 10 min to evaporate the acetone and then placed individually into 30 mL clear plastic portion cups (WNA Comet; Waddington North America, Chelmsford, MA). One cup containing the control filter paper and one containing the treatment filter paper were positioned at opposite ends of the tube. For each experiment, one tube received acetone-treated filter papers at both ends and served as a negative control. All assays were conducted in environmentally controlled rearing chambers maintained at 28 °C and 80% relative humidity. Mosquito distribution within the tube was recorded at 30 min following exposure. Percent repellency was calculated by using the following formula:


$$\begin{array}{c} \%\text{ Repellency }=\frac{\left({\text{N}}_{\text{Control}}-{\text{N}}_{\text{Treatment}}\right)}{\left({\text{N}}_{\text{Control}}+{\text{N}}_{\text{Treatment}}\right)}\times \text{1}\text{0}\text{0} \end{array}$$

where $N_{\text{control }}$and $N_{\text{Treatment}}$represent the number of mosquitoes present on the control and treatment sides of the tube, respectively. Replicates were excluded from analysis if a repellency % distribution in the negative control exceeded 30%.

### Statistical analysis

All statistical analyses and nonlinear regression modeling were performed using GraphPad Prism (version 10.2.3; GraphPad Software, San Diego, CA, USA). Statistical significance was assessed at α = 0.05 unless otherwise stated.

#### Topical toxicity of hemp leaf extract and CBD against adult female *Ae. aegypti*.

Dose–response relationships for topical adulticidal assays were analyzed by nonlinear regression using a log(dose)–response model with a variable slope (four-parameter logistic). Median lethal doses (LD_50_) and corresponding 95% confidence intervals (CI) were estimated from fitted models.

To compare the adulticidal activity of hemp leaf extract and CBD isolate at equivalent CBD doses (5 and 10 µg/mosquito), mortality data were first assessed for normality using the Shapiro–Wilk test. Because mortality data from CBD-treated groups deviated significantly from normality, pairwise comparisons between hemp extract and CBD isolate were conducted within each dose using the non-parametric Mann–Whitney U test.

#### Repellency of hemp leaf extracts and CBD against adult female *Ae. aegypti*.

For blood-feeding deterrence assays, repellency data were fitted using nonlinear regression (log[agonist] vs. normalized response; variable slope) to estimate effective doses producing 50% feeding inhibition (ED_50_ or EC_50_), along with 95% CI. To directly compare the repellency of hemp leaf extract and CBD isolate, hemp extract doses were converted to CBD-equivalent values based on prior chemical characterization [28]. Differences between fitted dose–response curves were evaluated using an extra sum-of-squares F-test.

For MART assays, differences between acetone control and each treatment dose were evaluated using a one-way ANOVA and Dunnett’s multiple comparisons test. Assumptions of normality and homogeneity of variance were evaluated using Shapiro–Wilk and Brown–Forsythe tests, respectively, and no major violations were detected.

**Ethics Statement:** This study did not involve human participants or live vertebrate animals. All experiments were conducted using laboratory-reared mosquitoes (*Ae. aegypti*, Liverpool strain). Defibrinated rabbit blood used for mosquito rearing and bioassays was obtained commercially from Hemostat Laboratories (Dixon, CA, USA). No ethical approval was required for this research.

## Results

### Topical toxicity of hemp leaf extract and CBD against adult female *Ae. aegypti*

**Fig 1:**
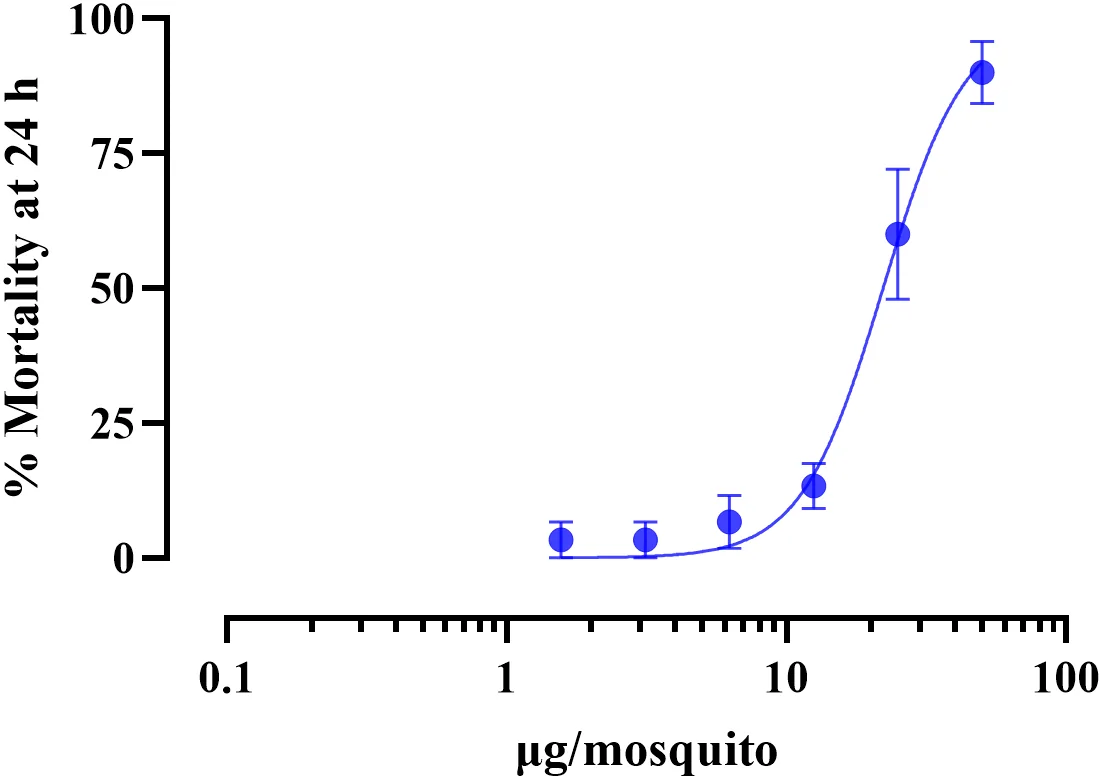
Dose–toxicity curve for topical application of hemp leaf extract to adult female *Ae. aegypti* at 24 h post-treatment. Each point represents mean percent mortality ± SEM from 3-6 replicates of 10 adult female mosquitoes per dose (1.56, 3.125, 6.25, 12.5, 25 or 50 μg of hemp leaf extract/mosquito). LD_50_ values and 95% CI were estimated from the fitted nonlinear regression model.

Topical application of hemp leaf extract to adult female *Ae. aegypti* resulted in dose-dependent mortality within 24 h post-treatment (Fig 1), but required high doses (LD_50_ = 22.13 µg, 95% CI = 18.86–26.18 µg). Across all replicates, the mean of the solvent control mortality was 3.3% ± 5.2%.

**Fig 2:**
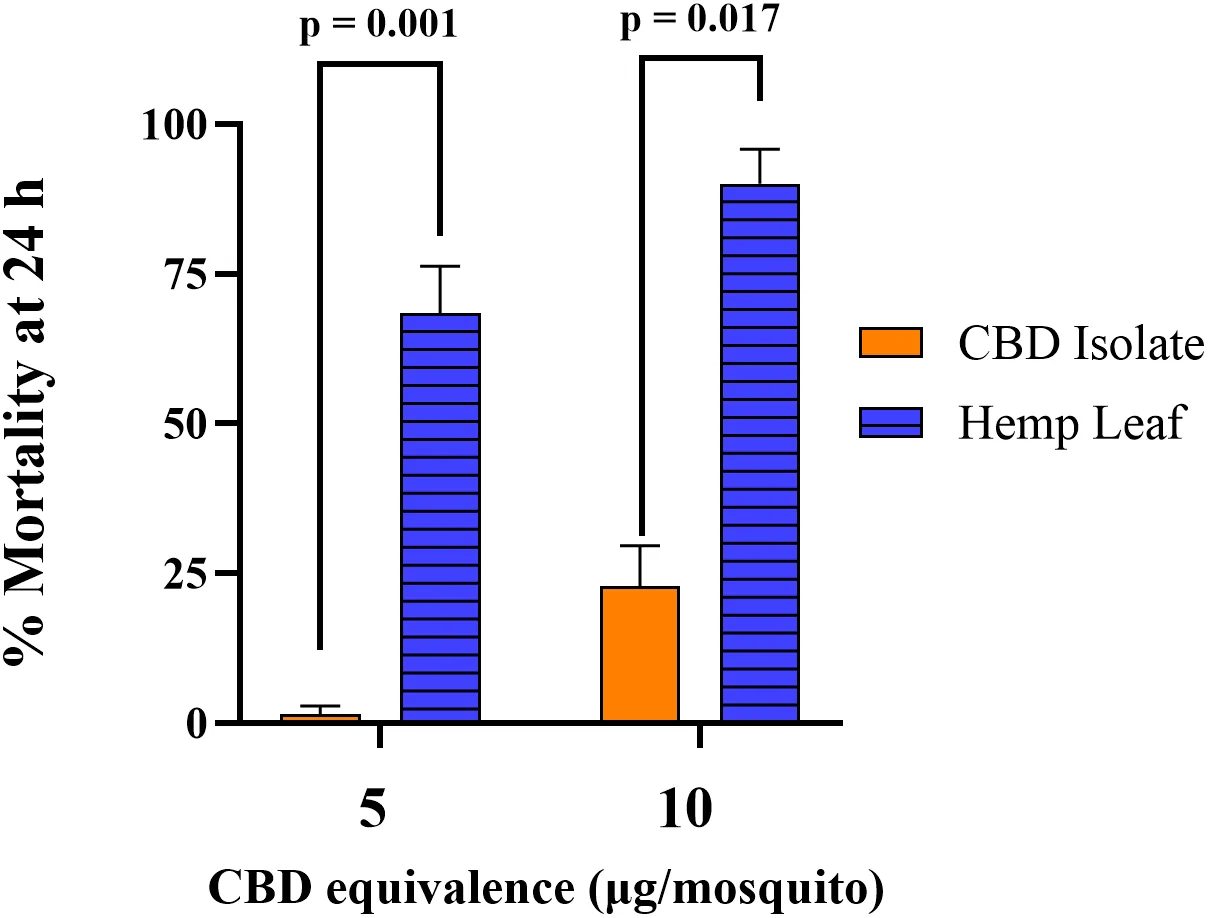
Topical adulticidal activity of hemp leaf extract and CBD isolate at equivalent CBD doses against adult female *Aedes aegypti.* Bars represent mean percent mortality (± SEM) at 24 h post-application across two CBD-equivalent doses: 5 µg/mosquito and 10 µg/mosquito, with corresponding hemp extract doses adjusted to deliver the same estimated amount of CBD. The data were analyzed using the non-parametric Mann-Whitney U test due to non-normal distribution in the CBD isolated group (Shapiro-Wilk, p < 0.0001). Mortality was significantly higher in mosquitoes treated with hemp extract compared to CBD at both 5 µg (p = 0.001, n = 6-7) and 10 µg (p = 0.017, n = 4-7).

To determine the proportion of the toxic activity of hemp leaf extract attributable to CBD, we tested the topical toxicity of a CBD isolate at doses equivalent to that found in the two highest doses of the leaf extract (i.e., 25 and 50 μg of this hemp leaf extract contain about 5 and 10 µg of CBD, respectively [28]). We found that CBD isolate at both doses induced substantially less mortality within 24 h than the corresponding doses of leaf extract (Fig 2). Across all replicates, the mean of the solvent control mortality was 4.29% ± 6.03%.

### Repellency of hemp leaf extracts and CBD against adult female *Ae. aegypti*

**Fig 3:**
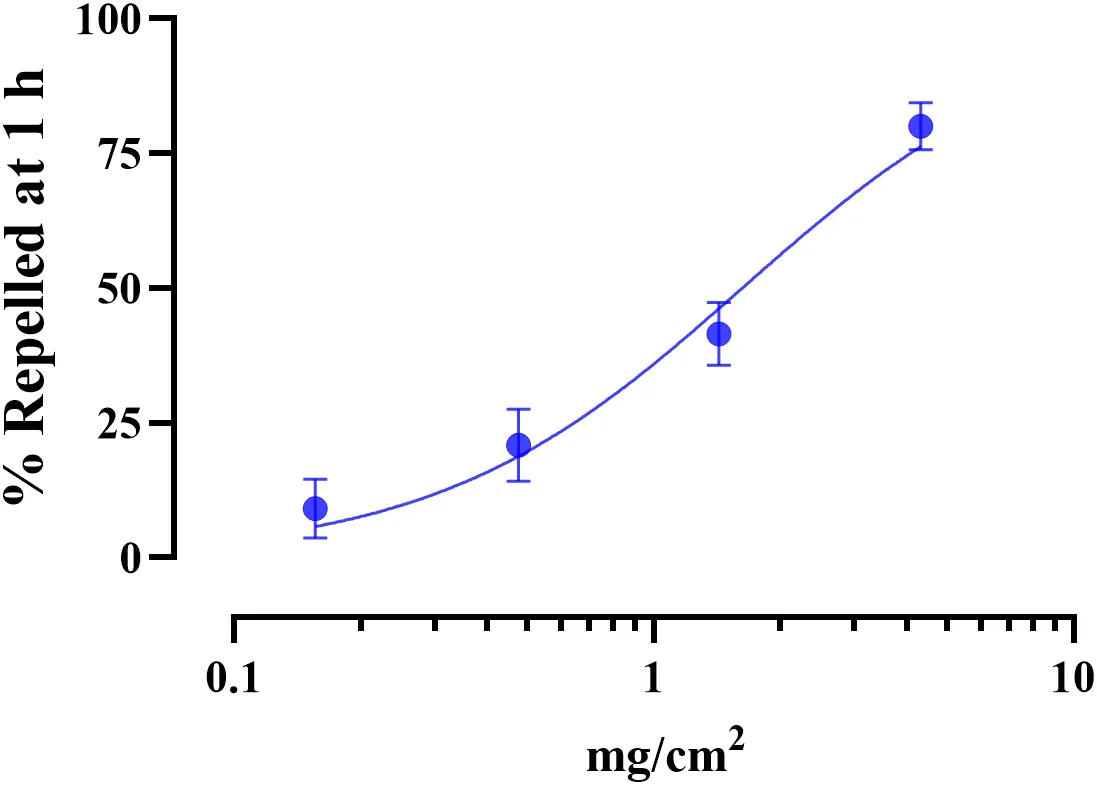
Dose -repellency curve for hemp leaf extract against blood-seeking adult female *Ae. aegypti* at 1 h. Each point represents mean percent repellency ± SEM from 7-8 replicates of 20 adult female mosquito per dose (0.15, 0.47, 1.42, and 4.33 mg of hemp leaf extract/ cm^2^). LD₅₀ values and 95% CI were estimated from the fitted nonlinear regression model.

Application of hemp extract to the membrane feeding discs resulted in dose-dependent repellency with an ED_50_ of 1.62 mg/cm^2^ (95% CI = 1.23–2.15 mg/cm^2^, Fig 3).

**Fig 4:**
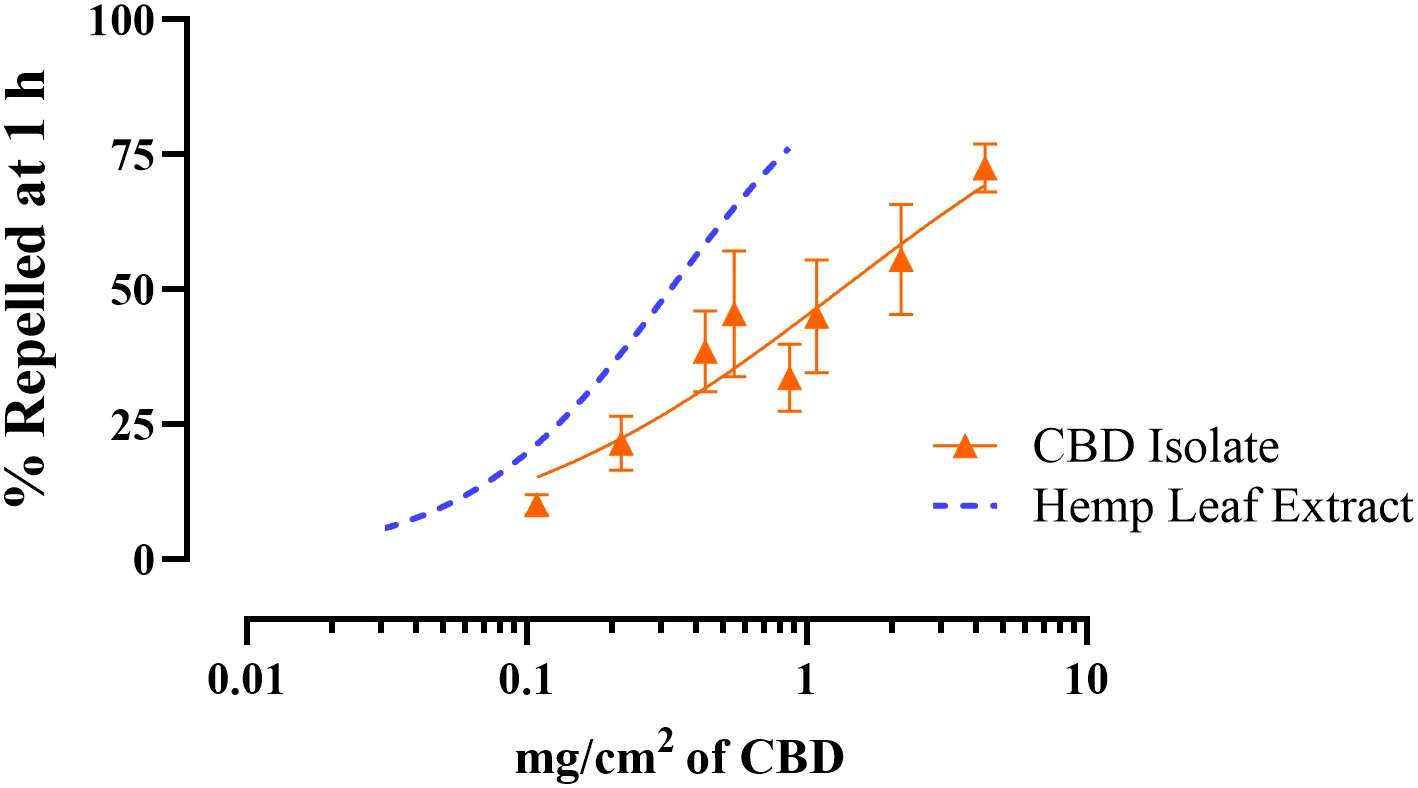
Repellency of CBD isolate against adult female *Ae. aegypti* evaluated through the no-choice membrane blood feeding assay. For reference, the dose-response relationship of the hemp leaf extract (Fig 3) is represented by the dashed blue line, with doses converted to their estimated CBD content (20% w/w) to allow for comparisons of potency. Each symbol represents mean ± SEM from 7-8 replicates of 20 adult female mosquito per dose. CBD isolate is a less potent repellent (EC_50_ = 1.321 mg/cm^2^) than hemp leaf extracts (ED_50_ = 0.325 mg/cm^2^).

The dose-repellency relationship of the CBD isolate (Fig 4) revealed an ED_50_ of 1.32 mg/cm^2^ (95% CI: 0.91–2.13 mg/cm^2^), which was ~ 4-times higher than that of the leaf extract when normalized for CBD content (0.325 mg/cm^2^ of CBD, 95% CI: 0.25–0.43 mg/cm^2^ of CBD). Notably, even when CBD was tested at doses over 6-times higher than that present in the highest dose of hemp extract, its maximal repellency remained lower than the extract (Fig 4.).

To determine if the repellency of the hemp leaf extract required contact, we used an airborne repellency test similar to that by Kwong et al [33]. No doses of hemp leaf extract showed significant repellency (Supplemental Fig 1). Due to lack of volatile repellency of the extract, the CBD isolate was not tested.

## Discussion

The toxicity assays in the present study revealed that hemp leaf extract applied topically to adult female *Ae. aegypti* induced dose-dependent mortality within 24 h. Compared to another plant extract (*Cinnamosma fragrans* bark) previously tested in our lab against the same strain of *Ae. aegypti*, the adulticidal potency of hemp leaf extract (22.13 µg/mosquito) was considerably weaker (LD_50_ for *C. fragrans* bark extract = 0.3 µg/mosquito [30]).

Consistent with the relatively weak adulticidal potency of hemp leaf extract, when adults were treated with CBD at equivalent doses found in hemp leaf extracts the mortality was much lower than that of the extract. This result suggests that CBD has minimal intrinsic topical toxicity to adult *Ae. aegypti* and likely does not contribute substantially to the adulticidal bioactivity of the extract under the conditions tested. This finding contrasts with our previous study on larvicidal activity of the same hemp leaf extract, which found that CBD was the principal larvicidal compound in the extract [28]. Given that CBD was by far the most abundant cannabinoid in the leaf extract [28], our results suggest that adulticidal activity likely reflect the contribution of constituents other than CBD, including minor cannabinoids, terpenoids, lipids or waxes,

In addition to cannabinoids, the hemp leaf extract consists of a complex chemical matrix that includes terpenes, plant waxes, and lipids, some of which may contribute to toxicity directly and/or by synergizing the toxicity of other compounds(s) [34,35]. Hemp-derived oils or complex extracts are known to be toxic to a diverse range of terrestrial arthropods, including aphids, spider mites, kissing bugs, and houseflies [23,36,37]. In these systems, the total bioactivity is often attributed to the collective effect of the plant’s secondary metabolites rather than a single compound. Future studies are needed to fractionate hemp extracts and oils to evaluate the contributions of non-cannabinoid components to the toxicity against adult female mosquitoes and other terrestrial arthropods.

The hemp leaf extract showed dose-dependent repellency in the blood-feeding assay. Compared to another plant extract (*C. fragrans* bark) previously tested in our lab using the same blood-feeding assay against the same strain of *Ae. aegypti*, the repellency of hemp leaf extract was considerably weaker (i.e., to reach ~75% repellency, *C. fragrans* bark extract only required 0.02 mg/cm^2^ vs. ~ 5 mg/cm^2^ needed for hemp leaf extract) [30]. Moreover, the hemp leaf extract showed no airborne repellency, suggesting that disruption of blood-feeding by hemp leaf extract requires contact, which aligns with previous studies in other insects. For example, Rothschild & Fairbairn (1980) found that *C. sativa* extracts deterred oviposition of the large white butterfly, *Pieris brassicae,* through contact, and Dadé et al. (2025), found that contact with *C. sativa* extracts repelled *Triatoma infestans* kissing bugs [37,38].

Although CBD isolate showed dose-dependent repellency of mosquitoes in the blood-feeding bioassay, the leaf extract was ~ 4-times more potent. Thus, similar to our conclusions from the topical toxicity bioassays, other compounds appear to play an important role in the repellency of the hemp leaf extract. Future studies are needed to evaluate the contributions of non-cannabinoid components to the repellency against adult female mosquitoes.

The exact mechanisms by which the hemp leaf extracts and CBD elicit toxic or repellent effects in adult mosquitoes remain unresolved. Because insects lack the endocannabinoid system found in mammals (CB_1_ and CB_2_ receptors), any toxic or repellent bioactivity of CBD must be mediated through interactions with alternative biochemical targets. Studies in mammalian systems have shown that CBD can modulate a broad array of biochemical targets, including various ion channels, G protein–coupled receptors, and acetylcholinesterase [39–43]. Whether orthologous targets are modulated in insects and contribute to toxicity or repellency is unknown. Further studies are needed to determine the effects of hemp extracts and CBD on the biochemistry and physiology of mosquitoes and other insects to understand how they elicit their toxic and repellent effects.

## Conclusions

This study demonstrates that hemp leaf extracts exhibit topical toxicity and repellency against the yellow fever mosquito, *Ae. aegypti*, and that these effects cannot be duplicated by CBD alone. The greater toxicity of the hemp leaf extract compared to the CBD isolate suggests important contributions by other compounds in the extracts. When repellency of the extract or CBD was evaluated, mosquitoes were only repelled when direct contact with the treated surface was allowed. Nevertheless, the hemp leaf extract was more effective than the CBD isolate, again pointing to the involvement of other compounds in adult repellency. Our findings advance our understanding of the potential uses of hemp and CBD against adult female *Ae. aegypti.* Identifying the additional components of the leaf extract that contribute to toxicity and repellency in adult female mosquitoes will be essential for evaluating the potential of hemp-based products in integrated mosquito management strategies. In addition, future research evaluating the human and environmental safety of using hemp-derived products as biopesticides is needed.

## Supporting information

S1 Tables Figures Dataset. (XLSX)

S1 Fig Airborne repellency of hemp leaf extract against *Ae. aegypti* at 30 min. Bars represent mean percent repelled (±SEM) after 30 min across 5 hemp leaf extract doses. Percent repellency at 30 min did not differ significantly among treatments (one-way ANOVA, F(5,37) = 1.19, p = 0.33).
(TIF)
